# Metacognitive Therapy versus Cognitive Behaviour Therapy in Adults with Major Depression: A Parallel Single-Blind Randomised Trial

**DOI:** 10.1038/s41598-020-64577-1

**Published:** 2020-05-12

**Authors:** Pia Callesen, David Reeves, Calvin Heal, Adrian Wells

**Affiliations:** 1 Cektos – Center for Kognitiv – og Metakognitiv Terapi, Riddergade 7, 1 sal, 4700 Næstved, Denmark; 2 University of Manchester, NIHR School for Primary Care Research, Manchester Academic Health Sciences Centre, Williamson Building, Manchester, M13 9PL UK; 3 University of Manchester, Centre for Biostatistics, Faculty of Biology, Medicine and Health, Manchester Academic Health Sciences Centre, Manchester, M13 9PL UK; 4 University of Manchester, School of Psychological Sciences, Faculty of Biology, Medicine and Health, Rawnsley Building, MRI, Manchester, M13 9WL UK; 5 Greater Manchester Mental Health NHS Foundation Trust, Manchester, UK

**Keywords:** Emotion, Human behaviour, Therapeutics, Outcomes research

## Abstract

In the last forty years therapy outcomes for depression have remained the same with approximately 50% of patients responding to treatments. Advances are urgently required. We hypothesised that a recent treatment, metacognitive therapy (MCT), might be more effective, by targeting mental control processes that directly contribute to depression. We assessed the clinical efficacy of MCT compared to current best psychotherapy practice, CBT, in adults with major depressive disorder. A parallel randomized single-blind trial was conducted in a primary care outpatient setting. This trial is registered with the ISCRTN registry, number ISRCTN82799488. In total 174 adults aged 18 years or older meeting Diagnostic and Statistical Manual of Mental Disorders IV criteria for major depressive disorder were eligible and consented to take part. 85 were randomly allocated to MCT and 89 to CBT. Randomisation was performed independently following pre-treatment assessment and was stratified for severity of depression (low < 20 vs high > =20) on the Hamilton Depression Rating Scale (HDRS) and on sex (male/female). Assessors and trial statisticians were blind to treatment allocation. Each treatment arm consisted of up to 24 sessions of up to 60 minutes each, delivered by trained clinical psychologists. The co-primary outcome measures were assessor rated symptom severity on the HDRS and self-reported symptom severity on the Beck Depression Inventory II (BDI-II) at post treatment. Secondary outcomes were scores six months post treatment on these measures and a range of symptom and mechanism variables. A key trial design feature was that each treatment was implemented to maximize individual patient benefit; hence time under therapy and number of sessions delivered could vary. Treated groups in the trial were very similar on most baseline characteristics. Data were analyzed on the basis of intention to treat (ITT). No differences were found on the HDRS at post treatment or follow-up (−0.95 [−2.88 to 0.98], p = 0.336; and −1.61 [−3.65 to 0.43], p = 0.122), but floor effects on this outcome were high. However, a significant difference favouring MCT was found on the BDI-II at post treatment (−5.49 [95% CI −8.90 to −2.08], p = 0.002), which was maintained at six-month follow-up (−4.64 [−8.21 to −1.06], p = 0.011). Following MCT 74% of patients compared with 52% in CBT met formal criteria for recovery on the BDI-II at post treatment (odds-ratio=2.42 [1.20 to 4.92], p = 0.014). At follow-up the proportions were 74% compared to 56% recovery (odds-ratio=2.19 [1.05 to 4.54], p = 0.036). Significant differences favouring MCT, also maintained over time, were observed for most secondary outcomes. The results were robust against controlling for time under therapy and when outcomes were assessed at a common 90 day mid-term time-point. Limitations of the study include the use of only two therapists where one treated 69% of patients, possible allegiance effects as the study was conducted in an established CBT clinic and the chief investigator is the originator of MCT and group differences in time under therapy. Never the less evidence from this study suggests that MCT had considerable beneficial effects in treating depression that may exceed CBT.

## Introduction

Depression is the second largest cause of global disability and is a source of major personal suffering, loss of quality of life and risk^1^. Antidepressant medications and psychological therapies are effective treatments. Of the available psychological therapies, cognitive behaviour therapy (CBT) is the most widely applied and studied. CBT is equal in effectiveness to Interpersonal therapy and is as effective in the short term as antidepressant medication^2,3^, but appears to have an advantage over antidepressant medication over longer term follow-up^4,5^. Despite this, only approximately 50 per cent of patients clinically improve in CBT and considerable relapse following treatment is an issue^6^. Thus, whilst CBT is the most strongly supported psychological treatment there have been no major advances in success rates for many years. Quite the contrary, there is evidence that the efficacy of CBT for depression is declining^7^.

Some researchers have attempted to augment CBT with methods aimed at relapse prevention, namely mindfulness-based interventions. Preliminary indications are that this might reduce relapse, but only in patients who have suffered at least three depression episodes^8^. However, this does not address the modest initial response rate seen in CBT amongst depressed patients, an area that commands improvement.

One potential advance may be a recent treatment that has proven highly effective in initial studies; metacognitive therapy (MCT)^9^. Metacognitive therapy differs from CBT in targeting specific psychological processes that are involved in the control of thinking thus enabling patients to free themselves from rumination and worry; cognitive processes central to depression. Small studies have examined the effects associated with MCT in depression. These suggest that MCT is associated with large reductions in depressive symptoms and that the gains are sustained over follow-up periods of 6–12 months. However, most of this evidence is from systematic case series and uncontrolled pre-post trials^10–13^. In a waiting-list controlled trial of adults with depression, MCT was superior to the control condition with 80% of patients recovered at post-treatment and 75% recovered at six months follow-up^14^. In another small-scale study that compared MCT and CBT, both were equally effective on primary depression measured at post treatment and follow up^15^. However, in a secondary analysis of these data patients who received MCT showed a superior outcome on measures of executive functioning at post treatment^16^. In a meta-analysis of anxiety and depression MCT was found more effective than no treatment control groups (g = 2.06) or CBT (g = 0.69)^17^.

In summary, MCT appears promising and might offer a necessary advance in depression treatment, but there is insufficient evidence at present from adequately powered trials to assess the relative efficacy of MCT compared with CBT in depression. The aim of the present study was to compare the effects of MCT against gold standard CBT in an appropriately powered trial of adult patients with major depressive disorder (MDD).

## Methods

### Study design and participants

We conducted a single-blind, parallel randomized trial of MCT versus CBT in an outpatient setting in a mental healthcare clinic in Næstved Denmark. Patients were recruited from referrals made by general practitioners to the clinic between January 2^nd^ 2011 and June 17^th^ 2015. Eligible patients were aged 18–70 years meeting diagnostic criteria for MDD assessed by an independent and experienced clinical psychologist using the structured clinical interview for DSM-IV-TR. Patients were excluded at initial assessment if receiving psychological therapy, suffering from psychosis, bipolar disorder, substance abuse, organic brain syndrome or learning difficulties, borderline personality disorder, had received earlier CBT or MCT, were pregnant and close to giving birth. An assessor, independent of the research team and blind to allocation administered the outcome measures at pre-treatment, post treatment and follow-up. The primary outcomes were the assessor-rated Hamilton Rating Scale for Depression (HDRS) and the self-rated Beck Depression Inventory II. A range of secondary measures were administered to assess anxiety symptoms and underlying psychological constructs (e.g. cognitive schemas and metacognitive beliefs). The Danish Regional research ethics committee for the region of Zealand granted approval for the study and all research was performed in accordance with ethical guidelines and clinical practice standards of Dansk Psykolog Forening. Informed consent was obtained from all participants prior to inclusion in the study. The study was not overseen by a separate data monitoring committee but data quality and completeness was monitored at quarterly held trial supervision meetings. The study was registered with the International clinical trials registry ISRCTN82799488 [4^th^ March, 2013].

### Randomization and masking

Following screening for eligibility, informed consent and the collection of baseline data, patients were sequentially randomly allocated to MCT or CBT according to computer-generated randomization lists independently produced by a trial statistician at the University of Manchester. Four lists were generated stratified by severity of depression as measured by the HDRS (<20 vs ≥20) and gender (male/female), each composed of randomized blocks of size 2 through 8. Allocation of each patient to a treatment condition was communicated to the trial administrator and the patient was assigned to one of two therapists depending on their availability. Treatment was open label but the independent assessors responsible for administering all measures were masked to patient allocation throughout the study. There were no reported instances of unmasking.

### Procedures

The CBT clinical protocols were published treatment manuals and NICE recommendations for the duration and nature of CBT interventions for depression^18,19^. Metacognitive therapy followed the standard published treatment manual with application of the disorder-specific depression model^9^. Patients were offered up to 24 sessions of CBT or MCT for depression. Sessions were delivered face to face and each lasted 60 minutes, decisions to terminate treatment were based on a patient achieving a score of ≤8 on the BDI II on two consecutive occasions and/or the therapist and patient agreeing on termination. Thus, the number of sessions delivered was determined by each patient’s response as would be the case in usual clinical practice. Each treatment was delivered by the same two therapists, both of whom were clinical psychologists and CBT therapists with a minimum of 10 years experience. Throughout the trial both therapists received CBT supervision from a CBT therapist with 35 years of CBT experience in Beck’s CBT (L. Holm). Supervision in MCT was provided by the originator of MCT (A. Wells). Supervision consisted of discussion of cases and observation of session video recordings. Before commencing the trial each therapist treated a series of training cases under supervision of the supervisors to become familiar with and skilled in the treatment protocols. Twenty-four patients (12 CBT and 12 MCT) cases were used in training.

### Interventions

Patients allocated to MCT received treatment that followed the published treatment manual^9^. MCT consisted of working with the therapist to generate a case conceptualization and shared understanding of the maintenance of depression based on the metacognitive model. Under this model depression is maintained by difficult to control repetitive thinking supported by unhelpful metacognitive beliefs. Treatment aims to improve cognitive-attentional control and modify such metacognitions. Treatment began by introducing the attention training technique (ATT) to enhance the sense of flexibility and control over thinking. ATT was practiced in-session throughout treatment. Subsequently detached mindfulness and rumination postponement experiments were introduced to foster an alternative relationship with trigger thoughts and to challenge metacognitive beliefs about uncontrollability of ruminative responses. Treatment also challenged negative beliefs about intractable causes of depression and metacognitions concerning the advantages of rumination and other maladaptive mind control strategies such as thought suppression, avoiding stress, and the use of rest and sleep to cope with thoughts and emotions. Throughout treatment the dialogue with patients utilized a meta-level Socratic discourse in which discussion centered on controlling reactions to thoughts rather than dealing with their content. Central to this process was the challenging of negative and positive metacognitive beliefs.

The CBT treatment was based on two published sources; Beck and colleagues (1979) treatment manual supplemented by Melanie Fennel’s protocol^18,19^. The overall aim of CBT was to help patients to identify and challenge negative automatic thoughts and core beliefs/assumptions and to enhance adaptive and pleasurable activity levels. The model is based on the principle that depression is maintained by negative and distorted interpretations of experience that occur as ‘automatic thoughts’ and that coupled with this individuals engage in coping behaviors that reduce opportunities for pleasure and mastery in life. These factors stem from underlying maladaptive beliefs about the self and world. In the first session a problem and goal list was agreed and patients were introduced to the CBT model using a critical situation and formulation from their current difficulties. At the end of the first session patients were given the booklet “coping with depression” to read^20^. In the following sessions negative automatic thoughts were identified and challenged using collaborative empiricism and behavioural experiments. Activity scheduling and mastery and pleasure techniques were introduced to counteract inactivity and inertia and this was maintained throughout therapy. Later in treatment the therapist worked with the patient to identify and challenge negative core beliefs about the self and world (e.g. “I’m a failure; “No one cares about me”). Homework assignments were used throughout.

Treatment quality, adherence and integrity were assessed using videotapes and written checklists of treatment sessions. Videotapes were screened by two independent and external clinical psychologists for potential contamination of methods. In addition 20 random videotapes of sessions (10 MCT, 10 CBT) stratified by therapist were selected and rated by 4 postgraduate psychology students for treatment adherence using adherence/fidelity checklists for CBT and MCT. Because one of the authors is the originator of MCT we were especially concerned that CBT be delivered optimally. Therefore we added an extra layer of quality assessment for CBT: a random sample of eight transcribed videotapes of CBT were sent to independent, leading practitioners in CBT and Fellows of the Academy of Cognitive Therapy (Dr Frank Dattilio and Dr Robert L. Leahy) for assessment of quality.

### Outcomes

A range of outcome and psychological process measures were collected at pre- and post-treatment and at longer-term follow-up (nominally 6 months post-treatment). Severity of depression symptoms was assessed using the Beck Depression Inventory (BDI-II, range 0–63) and the Hamilton Depression Rating Scale (HDRS, range 0–53)^21,22^. The BDI II was also administered at each treatment session to enable monitoring of patient progress. Anxiety symptom severity was assessed using the Beck Anxiety Inventory (BAI, range 0–63)^23^. Process measures were the Metacognitions Questionnaire 30 (MCQ-30, range 30–120)^24^, negative beliefs about rumination scale (NBRS, range 13–52)^25^, positive beliefs about rumination scale (PBRS, range 9–36)^26^, the rumination response scale, dysfunctional attitudes scale (DAS, range 40–280)^27^, and Young’s schema questionnaire- short version (YSQ-SF, range 75–450)^28^. We also computed reliable improvement and recovery rates based on the BDI-II: applying criterion ‘a’ of Jacobson and Truax^29^. A patient was classed as recovered if their BDI-II score was 11 or below at post-treatment and had reduced by 10 points or more from pre-treatment.

For evaluating the effectiveness of treatment we specified two primary endpoints for the trial: the Hamilton Depression Rating Scale (HDRS) and Beck’s Depression Inventory (BDI-II), both at the post-treatment time-point. These two primaries differ in that the BDI-II is patient self-report whilst the HDRS is assessor rated. Secondary endpoints were the BDI-II and HDRS at follow-up and the BAI and process measures at post-treatment and follow-up.

We evaluated patients’ expectations of the treatments they were receiving by administering two questions at treatment session 3: 1) How logical does the therapy you participate in appear to you? 0= not at all logical to 5 very logical; 2) How much do you believe the therapy will help you in reducing your symptoms? 0 = not at all to 5 = very much so. The quality of the therapist-patient relationship was also assessed at session 3 with Horvath and Green’s Working Alliance Inventory (WAI), that yields two scores; quality assessed by the patient and by the therapist^30^. The trial pack also contained attention and memory subscales of the Wechsler Adult Intelligence Scale intended for analysis in a subsequent study.

### Sample size calculation

At the time of trial planning there were no direct comparisons of MCT against CBT for depression to inform sample size estimates. We therefore planned the current study to detect a post-treatment difference between groups based on mean BDI-II scores observed in previous studies that tested MCT or CBT separately^6,12^. The mean following MCT was 10 and the mean for CBT 14, suggesting a group difference of 4 points. We used 7 as the standard deviation (SD)^12^ giving a standardized effect size of 0.57. Using an alpha of 5% and 90% power, this indicated that 64 participants per group were required. We increased the target sample by 20% to allow for pre-to-post treatment attrition.

## Data Analysis

Analysis was conducted in accordance with a pre-specified analysis plan - detailing the analytical models, primary and secondary outcomes, covariates, sensitivity analyses etc - finalised and formally signed-off before the final data-set was entered and analyzed. The primary analyses used intention-to-treat. For continuous outcomes a linear mixed effects regression model was applied incorporating all three time-points (pre-treatment, post-treatment and follow-up), and for binary outcomes (recovery) a logistic mixed effects model was used. Pre-specified covariates in the model were trial-arm, time-point, therapist, patient gender, baseline depression severity (HDRS < 20 versus HDRS > = 20), and use of psychiatric medication (yes/no). To these we added marital status, employment and education, which met protocol criteria for arm imbalance of more than 10% on one sub-category or more. Patient intercepts were specified as a random effect. The covariance matrix for the model was chosen as either unstructured or first order autoregressive depending upon whichever gave the lowest Bayesian Information Criteria score.

The effects of the intervention at post-treatment and follow-up were examined using the treatment group by time-point interaction terms from the mixed effects model analysis. No adjustments for multiple testing were applied and an alpha-value of 5% was used throughout. We ran three sensitivity analyses. First, nearly all outcome measures demonstrated skewness or kurtosis>1.0 at post-treatment or follow-up, therefore we validated statistical significance using a bootstrapped estimate of standard error, using 10,000 bootstrap samples. Second, we repeated the analyses using multiple imputation (MI) to impute missing values. There were very few missing values at baseline, therefore these were imputed by simple regression imputation using all available variables at baseline, but excluding treatment arm. MI was then used to impute missing outcome values at post-trial and follow-up, using the full set of variables and including the interaction term between treatment arm and time-point (for consistency with the analysis model). We used the chained-equations MI procedure and 20 MI datasets. Third, time durations from pre-treatment to post-treatment and from pre-treatment to follow-up varied between individuals, at least partly depending upon the number of sessions delivered. We therefore repeated the analyses including these time durations as a covariate to estimate the between-arm difference for a common duration time (the mean across arms).

We also ran two post-hoc analyses as further sensitivities against group differences in patterns of therapy delivery. First, a Cox regression of time-to-recovery from depression (in days since baseline), which estimates the point-ratio of group recovery rates regardless of chronological time (under the constant hazards assumption). This analysis controlled for the same set of covariates, with non-completers censored at their date of exit. Second, we compared BDI-II scores at a common time-point using each participant’s score at their therapy session closest to 90 days (the mid-point of the average treatment duration of 180 days). For participants who exited therapy or the study before 90 days we included their final measured BDI-II, but excluded two patients (one from each group) with no BDI-II score subsequent to baseline. All analysis was conducted using Stata version 14.

## Role of the funding source

There was no funding source for this study. All authors had full access to all the data in the study and AW had final responsibility for the decision to submit the publication.

## Results

**Figure 1:**
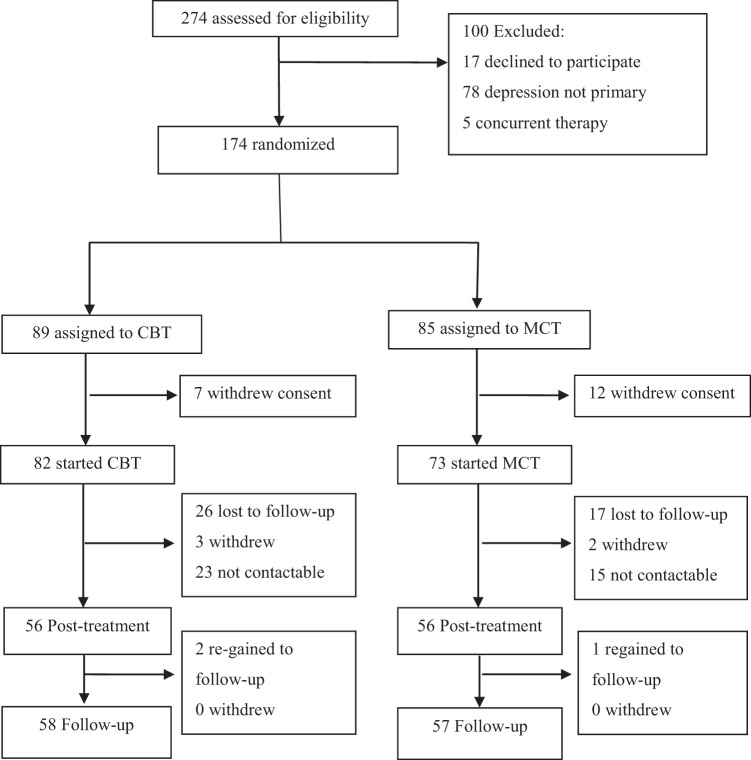
Trial Profile.

A total of 274 patients were assessed for study suitability following referral to the clinic for depression symptoms, of these 100 were excluded. Reasons for exclusion were as follows: 17 declined to participate, in 78 depression was not primary, 5 were receiving concurrent therapy. 174 were randomized, 89 to CBT and 85 to MCT, but 7 and 12 of these respectively withdrew consent (including use of their baseline data) after randomization. The remaining 155 patients (82 assigned to CBT and 73 assigned to MCT) participated in the trial (Fig. 1).

Table 1 provides demographic and clinical data for the sample at baseline which shows that the groups were well balanced at baseline on most variables. In both arms 69% of participants were female, the mean age was 35 years, and close to 70% were seen by one of the therapists. Rates of high depression severity were similar on the HDRS (72.5% versus 69.4%) as expected from stratification, but depression symptoms using the BDI-II were somewhat more severe in the MCT group, with 48% classified as severely/extremely depressed pre-treatment compared to 37% of the CBT group; and 17% had depression for 1 year or more compared to 12%. Half (50%) of the MCT group had middle-higher or university education compared to 31.3% of the CBT group. Out of 85 patients randomized to the MCT group, 57 (67.1%) were retained in the study up to 6 months follow-up, although the earlier post-treatment assessment was missing for one of these; while of 89 randomized to CBT, 58 (65.2%) completed the 6 months follow-up with post-treatment assessment missing for two.

**Table 1:** Baseline demographic and clinical characteristics.

	Cognitive Behaviour Therapy (n = 82)	Metacognitive Therapy (n = 73)
**Sex (stratification factor)**		
Male	26 (31.7%)	22 (30.1%)
Female	56 (68.3%)	51 (69.9%)
**Depression severity (stratification factor)**		
Low (HRSD < 20)	59 (72.0%)	51 (69.9%)
High (HRSD > = 20)	23 (28.0%)	22 (30.1%)
**Therapist**		
First therapist	57 (69.5%)	50 (68.5%)
Second therapist	25 (30.5%)	23 (31.5%)
Age	35 (13.4)	35 (11.9)
**Employment:**		
Employed	29 (35.4%)	35 (48.0%)
Sick leave	27 (32.9%)	22 (30.1%)
Student	13 (15.9%)	8 (11.0%)
Unemployed	8 (9.8%)	7 (9.6%)
Retired	5 (6.1%)	1 (1.37%)
**Education:**		
9^th^ Grade	7 (8.8%)	1 (1.4%)
High School	18 (22.5%)	13 (17.8%)
Short-higher (Business academies)	30 (37.5%)	22 (30.1%)
Middle higher (University colleges)/University	25 (31.2%)	37 (50.7%)
**Civil status:**		
Single	30 (36.6%)	19 (26.0%)
Married	27 (32.9%)	29 (39.7%)
In relationship	25 (30.5%)	25 (34.3%)
**Period of depression:**		
Under 1 year	71 (86.6%)	61 (83.6%)
1–2 years	7 (8.5%)	8 (11%)
2–3 years	3 (3.7%)	4 (5.5%)
More than 5 years	1 (1.2%)	0 (0.0%)
**Depression severity (based on BDI-II):**		
Mild mood disturbance	8 (9.9%)	2 (2.8%)
Borderline clinical	9 (11.1%)	10 (13.9%)
Moderate clinical	34 (42.0%)	26 (36.1%)
Severe	24 (29.6%)	27 (37.5%)
Extreme	6 (7.4%)	7 (9.7%)
**Other diagnoses**		
None	57 (69.5%)	51 (69.9%)
One or more	25 (30.5%)	22 (30.1%)
**On psychiatric medication**		
No	51 (62.2%)	49 (67.1%)
Yes	31 (37.8%)	24 (32.9%)
Beck Depression Inventory-II	28.2 (9.3)	29.6 (8.0)
Hamilton Depression Rating Scale	16.0 (5.0)	16.6 (5.1)
Beck Anxiety Inventory	17.2 (11.3)	16.5 (9.8)
Negative Beliefs about Rumination Scale	24.9 (7.3)	25.5 (7.4)
Positive Beliefs about Rumination Scale	21.1 (6.1)	21.2 (6.5)
Metacognitive Questionnaire 30	68.2 (14.0)	66.8 (14.1)
Dysfunction attitude scale	153.0 (32.4)	151.9 (35.4)
Young’s Schema Questionnaire- short version	252.1 (60.8)	248.2 (64.3)
Number of treatment sessions	6.7 (4.7)	5.5 (2.4)
Time in days from pre- to post-treatment assessments	194.8 (138.4)	163.9 (96.6)
Time in days from pre-treatment to follow-up assessments	394.3 (140.7)	373.3 (138.5)

Data are mean (SD) or n (%). HDRS = Hamilton Depression Rating Scale. BDI-II = Beck Depression Inventory-II.

For CBT the mean number of sessions delivered was 6.7 (SD = 4.7) with a range of 2–24, whilst for MCT it was 5.5 (SD = 2.4) with a range of 2–12. This difference was significant (difference = −1.2 [−2.4 to −0.02], p = 0.046). The mean interval between pre- and post-treatment measures was 194.8 days for CBT and 163.9 days for MCT though the difference was not significant (difference = 30.1 [−14.0 to 75.8, p = 0.176), as was the difference at follow-up (difference = 21.0 [−31.4 to 73.3], p = 0.429).

Rates of treatment completion (mutually agreed completion or termination of therapy) were 56 out of 73 (76.7%) in MCT and 56 out of 82 (68.3)% in CBT. Assessment at session 3 of patients’ judgements of how logical the treatments seemed to be showed no group difference (CBT M = 4.45, SD = 0.67; MCT M = 4.52, SD = 0.55), similarly session 3 expectancy of treatment being effective showed no difference (CBT M = 4.25, SD = 0.76; MCT M = 4.48, SD = 0.67). The quality of the therapeutic alliance at session 3 did not meet acceptable levels of skewness or kurtosis and was tested non-parametrically. There were no differences in patient (CBT M = 71.00, SD = 11.26; MCT M = 75.14, SD = 8.43) or therapist ratings (CBT M = 72.69, SD = 8.06; MCT M = 74.83, SD = 7.16). Ratings of treatment adherence were similar for each intervention and across the two therapists; CBT M = 86.5% and MCT M = 83%. Ratings of therapist competency in the CBT condition were undertaken by accredited CBT specialists Dr. Frank Dattilio and Dr. Robert Leahy who rated two full-length sessions and six 10-min extracts. Ratings were based on Blackburn’s Revised Cognitive Therapy Scale (CTSR) using five-point Likert scales. The ratings of therapist CBT competency were M = 85.6%. MCT competency was rated by the corresponding author on a subjective 0–100 scale and was based on overall performance as observed in supervision tapes and was M = 68%. Whilst direct comparisons are not possible the higher competency in CBT probably reflects the historical orientation and years of practise of the former therapy in the host clinic.

**Figure 2:**
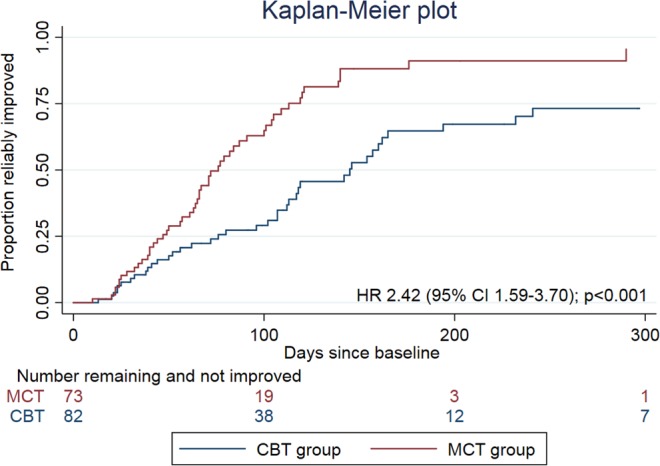
Kaplan-Meier Plot.

Results of the main analysis of primary and secondary endpoints are summarized in Table 2. There was no difference between groups at post-treatment on the HDRS primary outcome (−0.95 [−2.88 to 0.98], p = 0.336). However, there was a significant difference between groups on the primary endpoint of BDI-II at post-treatment, demonstrating superiority of MCT (−5.49 [95% CI −8.90 to −2.08], p = 0.002). The same pattern of results was repeated at the follow-up assessments (BDI-II −4.64 [−8.21 to −1.06], p = 0.011; HRSD −1.61 [−3.65 to 0.43], p = 0.122). Examining recovery on the BDI-II, the odds ratio for MCT compared to CBT was 2.42 [1.20 to 4.92], p = 0.014, at post treatment and 2.19 [1.05 to 4.54], p = 0.036, at follow-up, favouring MCT. The post-hoc Cox regression (Fig. 2) estimated a point rate of recovery under MCT across the therapy sessions of between two and three times the rate under CBT (HR = 2.57 [1.63 to 4.05], p < 0.001). Analysis of mid-term BDI scores included 81 CBT and 72 MCT participants; mean (SD) BDI-II scores were 17.2 (10.8) and 13.9 (11.6) respectively and the covariate-adjusted mean difference significantly favoured MCT (−4.82 [−8.07 to −1.57], p < 0.001).

**Table 2:** Summary of analyses of primary and secondary outcomes.

	CBT		MCT		Adjusted difference ^†^		Using bootstrapped SD	Adjusted difference using multiple imputation		Including time from baseline
	n	Mean (SD)	n	Mean (SD)	Mean (95% CI)	p-value	p-value	Mean (95%CI)	p-value	p-value
**Hamilton Depression Rating Scale**										
Post-treatment*	54	2.93 (3.88)	55	2.67 (4.21)	−0.95 (−2.88,0.98)	0.336	0.357	−2.08 (−4.70,0.54)	0.119	0.446
Follow-up	58	3.74 (4.98)	57	2.67 (2.87)	−1.61 (−3.65,0.43)	0.122	0.126	−1.99 (−4.77,0.79)	0.159	0.228
**Beck Depression Inventory-II**										
Post-treatment*	56	8.14 (8.62)	56	4.14 (4.97)	−5.49 (−8.90,−2.08)	0.002	0.002	−6.43 (−10.96,−1.91)	0.006	0.001
Follow-up	55	7.67 (10.20)	54	4.13 (5.24)	−4.64 (−8.21,−1.06)	0.011	0.015	−5.61 (−10.83,−0.40)	0.035	0.025
**Beck Anxiety Inventory**										
Post-treatment	53	6.08 (6.31)	53	3.78 (4.11)	−1.51 (−4.70,1.67)	0.352	0.384	−2.52 (−6.62,1.57)	0.226	0.388
Follow-up	50	5.28 (7.30)	50	3.18 (4.04)	−0.91 (−4.11,2.30)	0.579	0.601	−2.48 (−6.92,1.95)	0.271	0.730
**Recovery on the BDI-II**^**‡**^										
Post-treatment (%, n)	82	52%, 43	73	74%, 54	OR = 2.42 (1.20,4.92)	0.014	0.031	NA	NA	0.017^§^
Follow-up (%, n)	82	56%, 46	73	74%, 54	OR = 2.19 (1.05,4.54)	0.036	0.068	NA	NA	0.045^§^
**Metacognitive Questionnaire 30**										
Post-treatment	47	53.15 (13.21)	50	43.6 (10.97)	−9.97 (−15.40,−4.54)	<0.000	0.003	−8.22 (−14.80,−1.63)	0.015	0.001
Follow-up	46	49.41 (14.54)	48	44 (11.02)	−5.81 (−11.19,−0.43)	0.034	0.103	−6.46 (−13.53,0.60)	0.073	0.071
**Negative Beliefs about Rumination Scale**										
Post−treatment	47	17.91 (4.96)	51	17.27 (4.95)	−1.82 (−4.13,0.48)	0.080	0.115	−1.99 (−5.17,1.18)	0.217	0.120
Follow-up	46	18.30 (6.28)	49	17.0 (4.91)	−2.13 (−4.47,−0.22)	0.024	0.042	−2.95 (−6.20,0.30)	0.075	0.075
**Positive Beliefs about Rumination Scale**										
Post−treatment	47	14.43 (6.44)	51	9.98 (1.98)	−4.67 (−7.3,−2.08)	<0.001	0.001	−4.31 (−7.20,−1.43)	0.004	0.001
Follow-up	47	14.04 (5.97)	49	10.61 (2.60)	−3.49 (−5.95,−1.03)	0.006	0.011	−3.81 (−6.60,−1.02)	0.008	0.013
**Dysfunctional Attitude Scale**										
Post-treatment	47	114.40 (39.13)	50	96.98 (36.99)	−16.34 (−29.42,−3.26)	0.014	0.024	−18.53 (−33.77,−3.29)	0.017	0.029
Follow-up	47	112.77 (37.61)	48	93.83 (36.10)	−18.55 (−31.16,−5.95)	0.004	0.009	−21.08 (−39.75,−2.41)	0.027	0.012
**Young’s Schema Questionnaire**										
Post-treatment	46	177.83 (59.51)	50	159.8 (54.67)	−16.00 (−36.93,4.92)	0.134	0.178	−20.67 (−46.99,5.65)	0.123	0.127
Follow-up	47	173.26 (66.53)	48	156.02 (53.42)	−11.71 (−33.35,9.94)	0.289	0.336	−21.49 (−50.80,7.81)	0.149	0.518

*Primary outcome. ^†^Group difference from mixed effects regression, controlling for therapist, patient gender, baseline depression severity, use of psychiatric medication, marital status, education and employment. ^‡^Non-completers assumed to be fails. ^§^Non-completers assigned sample mean durations. NA = not applicable (the primary analysis includes all cases). BDI-II = Beck Depression Inventory-II.

We found that MCT was not different from CBT with regard to anxiety (BAI) at either post-treatment (−1.51 [−4.70 to 1.67], p = 0.352) or follow-up (−0.91 [−4.11 to 2.30], p = 0.579). However, there were differences favouring MCT at both post treatment and follow-up on metacognitive belief scores (−9.97 [−15.40 to −4.54, p < 0.001; and −5.81 [−11.19 to −0.43], p = 0.034), positive beliefs about rumination (−4.67 [−7.30 to −2.08], p < 0.001; and −3.49 [−5.95 to −1.03], p = 0.006), negative beliefs about rumination (−1.82 [−4.13 to −0.48], p = 0.080; and −2.13 [−4.47 to −0.22], p = 0.024), and dysfunctional attitudes (−16.34 [−29.42 to −3.26, p = 0.014; and −18.55 [−31.16 to −5.95], p = 0.004). Differences were not evident on the Young Schema Questionnaire (−16.00[−36.93 to 4.92, p = 0.134; and −11.71 [−33.35 to 9.94], p = 0.289).

Sensitivity analysis changed the statistical significance of the results for three secondary outcomes, all of which ceased to be statistically significant (p >0.05) under one or more of the three sensitivity analyses: reliable improvement on the BDI-II, meta-cognitive beliefs and negative beliefs about rumination, all at follow-up.

Safety and adverse events (increased suicidality, death, self-injury) associated with the treatments were monitored throughout the trial. There were no adverse events reported. We had intended to assess the impact of treatment on rumination measured with the RRS, but a clerical error led to the version included in the trial assessment pack being incomplete.

## Discussion

We found that MCT was superior to CBT at post treatment and follow-up on the primary outcome of depression symptoms as measured using the BDI-II but treatments did not differ significantly on the HDRS. MCT was superior at both time-points on the majority of secondary outcome measures. The majority of findings were robust under sensitivity analyses accounting for missing values, non-normal distributions, and variability in intervals between measurement points; h exceptions were reliable change on BDI-II, metacognitive beliefs and negative beliefs about rumination, where group differences at follow-up ceased to be statistically significant (p >0.05).

The potential clinical importance of the treatment effects can be assessed by the relative recovery rates across the treatment conditions as measured by the BDI-II: 74% of MCT participants and 52% of CBT participants met criteria for recovery at post treatment, while at follow-up the proportions were 74% and 56% respectively.

Relative to CBT, MCT appears to be associated with a greater reduction in depression symptoms on the BDI-II but not on the HDRS. These differential results may plausibly be related to floor effects on the HDRS: at post-treatment 54% of the CBT group and 58% of the MCT group had an HDRS score of zero or 1, greatly limiting the variability for detecting a difference. That is, HDRS scores appear to have bottomed out in both groups prior to post-treatment assessment. Unfortunately, no interim HDRS measures were taken that might confirm this. It may also be the case that the BDI captures different sets of depression symptoms than the HDRS. For example, the HDRS has been criticised due to a multifactorial structure in which some items are poor measures of depression severity^31^.

The number of treatment sessions delivered depended partly upon the rate at which depression symptoms declined in a patient, and MCT patients received around one fewer therapy sessions on average and recovered over the sessions at a rate more than double that under CBT. This finding could have important implications, suggesting that MCT might be delivered more cost-effectively.

### Strengths and limitations

This trial has strengths and limitations. It was larger than previous studies of MCT and was conducted in a context making it applicable to typical primary-care clinical settings. We used pre-published treatment manuals and took major steps to ensure treatment adherence, fidelity and competency of therapists. A planned rumination measure had to be excluded due to clerical error resulting in the trial pack version being incomplete. Only two therapists were involved, therefore the findings require validation on a larger therapist group. One therapist treated more patients than the other because of time needed for maternity leave during the course of the trial. This may reduce the generalisability of the effects observed as one clinician treated the majority of the patients. Initially planned collection of mid-study outcome measures was abandoned out of concern over participant burden, but to partially compensate we analysed BDI-II session scores collected closest to the mid-point.

Although the clinicians were highly experienced in the delivery of CBT with a proven track-record in this approach, they were much less experienced in the delivery of MCT. On the one hand this may have limited their ability to deliver MCT to maximum effectiveness, but on the other the treatment novelty may have increased motivation thereby improving outcomes. The variability in the time course over which treatment was administered reflects standard clinical practice but is a key factor that may have impacted on outcomes. However, the CBT group on average experienced a higher number of treatment sessions, which would be likely to reduce any group differences, and sensitivity analyses controlling group differences in the duration of treatment and assessment of mid-term BDI did not change the results. The initiator of this research study (A. Wells) is the originator of MCT which may have introduced bias. Therefore, further, fully independent research is required to validate and extend the current findings to other populations and service settings.

Whilst the treatment groups appeared well balanced in most respects, more MCT patients withdrew consent immediately following randomization (12 compared to 7 under the CBT condition). The difference is not statistically significant (Fisher’s exact test, *p* = 0.228), but conceivably some patients (in both groups) were concerned about not receiving the treatment they most desired, though unfortunately we lack actual data on reasons. In addition, we were unable to include these individuals in the ITT analysis, and this may have resulted in a small degree of bias in our effect size estimates.

### Comparison with other studies

The results of the present study compare meaningfully with other outcome studies (mainly on anxiety). In a meta-analysis of the effects of MCT on anxiety and depression the controlled effect size for MCT was Hedges g = 2.06 and MCT was more effective than CBT (g = 0.68)^17^. Recovery rates of 75–80% at post treatment and 66–75% at 6 month follow-up amongst depressed patients following MCT have been reported in smaller studies^10,15^, The rates in the present study of 74% at both post treatment and follow-up concur closely with these. In the case of CBT the results we obtained of 53% recovery at post treatment and 57% at follow-up align with other published studies; where 50% response rates are a good outcome in CBT^5^. In fact our CBT follow-up rates are slightly better than those typically reported and this effect may reflect our optimization of treatment to match patient need.

We observed differences on most of our secondary process outcomes. Of particular interest, participants receiving MCT improved more in measures of both metacognition and dysfunctional beliefs (i.e. attitudes). We interpret this as indicating that MCT produces wider ranging changes in thinking. There is likely to be overlap in mechanisms of change in MCT and CBT. Studies in the area of anxiety have shown that CBT can lead to changes in metacognitions and change in this variable is a more robust predictor of treatment response than change in cognition^32^. This area is yet to be investigated in depression, but it seems possible that treatment-specific patterns of change in metacognitive regulation as hypothesized in MCT^33^ may underpin the greater effects observed.

## Supplementary information

Supplementary information

Supplementary information2

## Data Availability

The datasets generated during and/or analysed during the current study are available from the corresponding author on reasonable request.
