# Author Correction: Metacognitive Therapy versus Cognitive Behaviour Therapy in Adults with Major Depression: A Parallel Single-Blind Randomised Trial

**DOI:** 10.1038/s41598-020-68559-1

**Published:** 2020-07-07

**Authors:** Pia Callesen, David Reeves, Calvin Heal, Adrian Wells

**Affiliations:** 1 Cektos – Center for Kognitiv – Og Metakognitiv Terapi, Riddergade 7, 1 sal, 4700 Næstved, Denmark; 2 University of Manchester, NIHR School for Primary Care Research, Manchester Academic Health Sciences Centre, Williamson Building, Manchester, M13 9PL UK; 3 University of Manchester, Centre for Biostatistics, Faculty of Biology, Medicine and Health, Manchester Academic Health Sciences Centre, Manchester, M13 9PL UK; 4 University of Manchester, School of Psychological Sciences, Faculty of Biology, Medicine and Health, Rawnsley Building, MRI, Manchester, M13 9WL UK; 5 Greater Manchester Mental Health NHS Foundation Trust, Manchester, UK

Correction to: *Scientific Reports* https://doi.org/10.1038/s41598-020-64577-1, published online 12 May 2020

In Figure 2, the hazard ratio (HR) and 95% Cl were incorrect as they do not control for covariates. The correct Figure 2 appears below as Figure 1.

**Figure 1:**
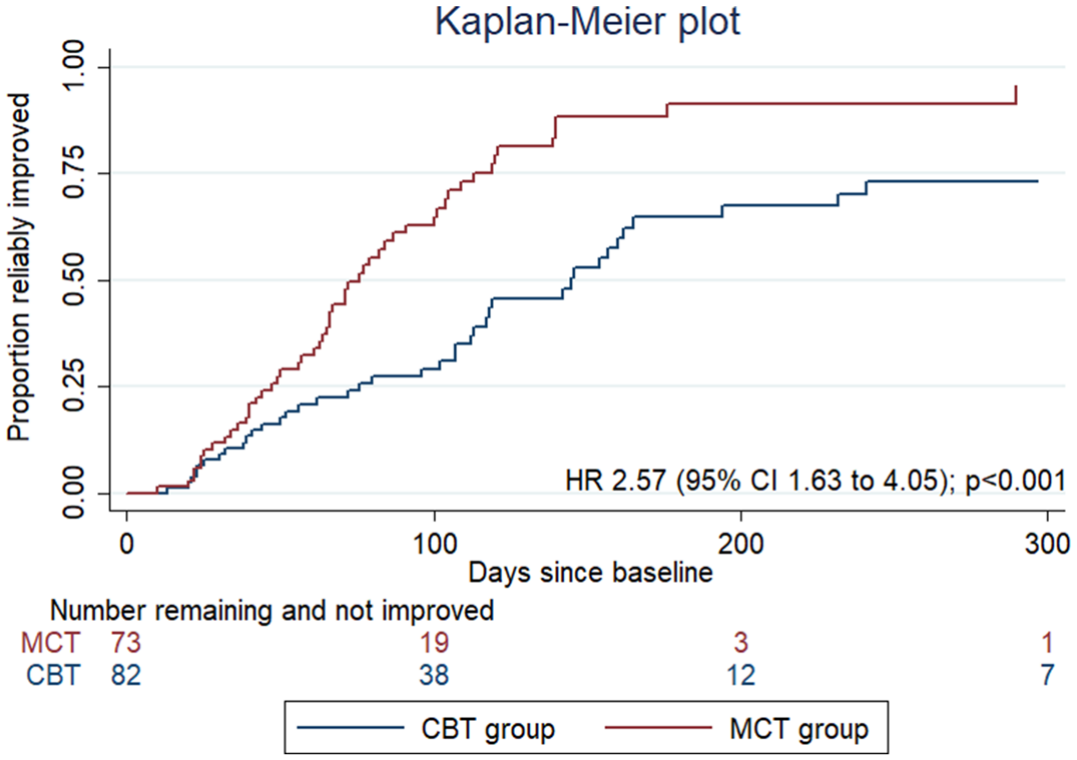
Kaplan-Meier Plot.

