# Supplementary material for: Metacognitive Therapy versus Cognitive Behaviour Therapy in Adults with Major Depression: A Parallel Single-Blind Randomised Trial
Source: Sci Rep. 2020 May 12;10:7878. doi: 10.1038/s41598-020-64577-1 (PMC7217821; doi:10.1038/s41598-020-64577-1)
Supplement: Supplementary file 2 — Supplementary information2 [file 41598_2020_64577_MOESM2_ESM.pdf]

Protocol: version 3 [13-09-2019]

Metacognitive Therapy versus Cognitive Behaviour Therapy in Adults with Major Depression: A Parallel Single-Blind Randomised Trial

Chief Investigator:

Prof. Adrian Wells

Co-investigator:

Dr. Pia Callesen

Ethical Approval: Research Ethics Committee for Region Zealand (Denmark) SJ-201 (notification no. 27761)

Sponsor: The University of Manchester, UK

Trial Registration: ISRCTN82799488 [04-03-2013]

ISRCTN82799488 https://doi.org/10.1186/ISRCTN82799488

Table of Contents

1. LAY SUMMARY……………………………………………………………………………3

2. BACKGROUND AND RATIONALE………………………………………………………3

3. STUDY AIMS AND MEASURES…………………………………………………………..5

4. RESEARCH HYPOTHESES…………………………………………………………….......6

5. METHODS……………………………………………………………………………….......6

   5.1. Participants and sampling procedure……………………………………………………6

   5.2. Inclusion criteria…………………………………………………………………………6

   5.3. Exclusion criteria……………………………………………………………………...7

6. OUTCOME MEASURES……………………………………………………………………7

   6.1. Primary outcome measures……………………………………………………………..7

   6.2. Secondary outcomes measures…………………………………………………………8

7. ETHICAL CONSIDERATIONS………………...11

8. SAFETY REPORTING AND ADVERSE EVENTS………………………………………11

9. TRIAL DESIGN AND ANALYSIS…………………………………………………………..12

   9.1. Sample size………………………………………………………………………………13

   9.2. Randomization and data analysis……………………………………………………...13

10. BLINDING…………………………………………………………………………………...13

11. INTERVENTIONS…………………………………………………………………………..14

   11.1. Cognitive behaviour therapy…………………………………………………………...14

   11.2. Meta-cognitive therapy…………………………………………………………………14

12. COMPETENCY AND TREATMENT ADHERENCE……………………………………...14

13. REFERENCES……………………………………………………………………………….16

APPENDIX 1: Study flow chart……………………………………………………………….21

APPENDIX 2: Schedule of measurement……………………………………………………..22

1. Lay Summary

Major Depressive Disorder (MDD) is one of the largest health problems worldwide. Psychological treatments show variable outcome rates with approximately 50% recovery in ‘gold standard’ cognitive behaviour therapies (CBT’s). More effective treatments for depression are needed. Metacognitive therapy (MCT) is a newer treatment for MDD that has produced promising results in preliminary studies. Hagen et al (submitted) showed very large effect sizes in a randomised trial comparing wait-list with MCT. In their study 70-80% could be classified as recovered. The next step is to compare MCT with CBT in a definitive trial.

In this study a large parallel randomised single-blind trial will be conducted comparing metacognitive therapy (MCT) and Cognitive Behavioural Therapy (CBT). Adult patients diagnosed with major depressive disorder (MDD) will be assigned to treatment consisting of up to 24 sessions of either CBT or MCT and the treatment terminated when remission is reached defined as two consecutives scores of ≤8 on Beck’s Depression Inventory II (BDI-II). Patients will be followed-up for 6 months post-treatment.

2. Background and Rationale

Depression is considered one of the largest health problems around the world. Several different medical and psychological treatments have been developed to treat the condition with mixed effects. Therapies for depression involve interpersonal therapy (Cuijpers, Geraedts, et al., 2011), Brief psychodynamic therapy (Driessen et al., 2015) and mindfulness-based therapies (MBSR and MBCT, (Gotink et al., 2015).

The most widely tested psychological treatment for MDD is cognitive behaviour therapy (CBT). CBT was first introduced by Dr Aaron T Beck (A. T. Beck, Rush, A.J., Shaw, B. F., & Emery, G. , 1979) and involves cognitive and behavioural strategies including restructuring of negative and biased thoughts and beliefs that the individual holds about the self, others and the future. Furthermore, CBT for MDD involves increasing levels of mastery and pleasurable activities. In CBT theory depression is caused by activated negative schemas (beliefs) e.g. "I'm unlovable" which colour the interpretation of experience and result in low mood.

The effect of CBT on MDD has been studied across multiple randomised controlled trials (RCT's) with moderate effect sizes (Cristea et al., 2015). A recent meta-analysis, however, reported that the effects of CBT as an antidepressant treatment has declined over the years (Johnsen & Friborg, 2015) suggesting that better treatments for depression are needed in order to enhance treatment outcome for this population.

A review of the literature shows that the majority of randomised trials give recovery rates of approximately 50% in ITT analysis for different psychological treatments. These include CBT, interpersonal therapy, antidepressant medication and supportive therapy. However, there are a number of biases, which make comparisons and conclusions challenging. These include treatment integrity, treatment duration and the lack of a valid and objective definition of treatment outcome and clinically significant change. The present trial is designed to overcome these biases and test MCT versus CBT in a definitive trial.

Metacognitive therapy (MCT) is a more recent treatment for depression. MCT is based on the Self-Regulatory Executive Function model (S-REF model) developed my Wells & Mathews (1994; 1996). According to this model psychological disorders are not caused by negative schemas but instead result from a style of thinking called the Cognitive Attentional Syndrome (CAS). In depression the CAS consists of voluntary mental coping strategies dominated by rumination/worry, avoidance, and mood monitoring along with other strategies that backfire and result in depressive symptoms. The cognitive attentional syndrome is driven by metacognitive beliefs such as the belief that rumination is a useful means of finding an answer to depression and the belief that depressive thinking is uncontrollable.

To date metacognitive therapy (MCT) for depression has been tested in a limited number of studies that have shown MCT to be a promising intervention that may improve on recovery rates. For example, Dammen et al (2015) implemented a group MCT intervention on 11 depressed individuals and found that 91% of them were recovered at follow-up (Callesen, Jensen, & Wells, 2014; Dammen, Papageorgiou, & Wells, 2015; Wells et al., 2009).

Jordan et al. (2014) conducted a randomised trial in New Zealand in which 48 depressed patients were randomised to either CBT or MCT. Effect sizes were moderate-to-large for both treatments

with no significant differences on primary measures. The number of participants meeting response criteria for each condition was slightly better in MCT (52%) than CBT (44%). In addition, follow-up analysis revealed that MCT produced greater changes in measures of improved executive control and spatial working memory (Groves et al., 2015). Several limitations were found in this study. First of all, Training or supervision in MCT was limited and post-hoc analyses suggested that MCT might have been adversely affected by comorbidity as 48% were suffering from social anxiety in the MCT condition compared to 28% in the CBT condition.

Hagen et al (submitted) conducted a RCT comparing MCT to a wait-list condition. Results from this trial demonstrated very large effect sizes for MCT. In this trial thirty-nine patients were randomly assigned to immediate MCT (10 sessions) or to a 10-week wait list period (WL). The WL-group received 10 sessions of MCT after the waiting period. Participants receiving MCT improved significantly more than the WL group with very large controlled effect sizes for both depressive (d = 2.51) and anxious symptoms (d = 1.92). Approximately 70-80% could be classified as recovered on the BDI-II at post-treatment and 6 months follow-up following immediate MCT, whilst 5% of the WL patients recovered during the waiting period. These findings suggest that a non-hybrid, pure version of MCT may be particularly effective.

The current proposed trial is designed to test MCT for MDD by comparing it to a bona fide benchmark treatment; CBT. The trial utilises a strict methodology intended to reduce potential biases encountered in earlier studies. Patients are allocated to a total possible number of treatment sessions with pre-set criteria for potential early termination. They receive up to 24 sessions of therapy or until they reach a predetermined agreed recovered state. Strict session-by-session manuals for both treatments, expert supervision in both CBT and MCT and objective definitions of recovery and relapse will be adopted using a conservative and clinical change index as defined by Jacobson and Truax (1991).

3. Study Aims and Measures

The aims of the present study are as follows:

1. To assess the relative efficacy of MCT versus CBT in the treatment of patients with major depressive disorder. Primary outcome variables are: Becks Depression Inventory –II (BDI-II) and Hamilton Depression Rating Scale (HDRS)
2. To assess the relative efficacy of MCT vs. CBT in the treatment of patients with major depressive disorder on secondary outcome variables: Becks Anxiety Inventory (BAI), process variables (Rumination time measured by MDD-S) and negative/positive metacognitive beliefs (NBRS/PBRS) as well as schema-related content variables (The Dysfunctional Attitude Scale – (DAS)) will be used.
3. To determine if treatment effects are maintained over a 6 months follow-up period.
4. To investigate recovery rates and clinical significant change levels in CBT and MCT
5. To assess dropout rates, treatment adherence, and the quality of the therapeutic alliance in both treatment arms in order to detect any potential differences in MCT and CBT.

4. Research hypotheses

The null hypothesis predicts that there will be no significant differences in primary and secondary outcome variables or recovery rates in MCT and CBT at post treatment and at follow-up.

5. Methods

5.1. Participants and sampling procedures

Patients referred from their general practitioner (GPs) with a diagnosis of depression to a large Danish outpatient facility called CEKTOS (Center for Kognitiv Terapi) will be assessed for eligibility. In Denmark the NHS is partly funded by the state and partly by the patients themselves. Therefore, a payment of 315.00 Danish kr. pr. session will be administered for all participants in the trial. The study flow chart is presented in appendix 1.

5.2. Inclusion criteria

- Patients from 18-70 years of age with a main or primary diagnosis of major depressive disorder (MDD) according to the Structured Clinical Interview for DSM-IV-TR, Axis I Disorders (SCID-I)
- Patients who agree to attend therapy in the trial and have not received other psychological treatments for the current depressive episode.
- Patients in combined medical treatment as long as they are stable or willing to remain stable on medication
- Patients who are willing and able to sign the informed consent

5.3. Exclusion criteria

- Patients suffering from psychosis, substance abuse or bipolar disorder as determined by SCID I
- Patients suffering from borderline personality disorder as screened by SCID II
- Patients with organic brain syndrome or learning difficulty
- Female patients who are pregnant and close to giving birth
- Patients who have not responded favourably to a previous trial of either CBT or MCT

6. Outcome measures

A battery of assessments will be administered by an independent assessor blind to treatment allocation at pre-treatment, post-treatment and at 6- months follow-up. The primary outcome measures are Beck's Depression Inventory (BDI-II) and the Hamilton Depression Rating Scale (HDRS). We chose both a self-rating scale and an interview-based scale in order to account for effects in different ways. In addition, several secondary self-report measures will be administered at pre-treatment, post-treatment and follow-up. The schedule of measures is presented in appendix 2:

6.1. Primary outcome measures:

Depressive symptoms (Beck Depression Inventory – BDI-II)

The BDI was developed by Beck and his colleges in 1961 and has become one of the most widely used instruments for assessing the intensity of depression. The inventory has been revised several times and the most recent version is Becks Depression Inventory-II (A. T. Beck, Steer, R.A., Ball, R., & Ranieri, W., 1996). The BDI-II is a widely used reliable instrument to assess the severity of depressive symptoms. In fact, the BDI-II shows much better internal reliability than the BDI-I (Dozois, Dobson, & Ahnberg, 1998).It is a self-report inventory consisting of 21 items, where symptoms such as sadness, loss of pleasure, changes in sleep pattern, and worthlessness are measured and rated on a four-point scale ranging from 0 (not present) to 3 (severe). All the scores are summated to form a total score, where the maximum score obtainable is 63. A score of 0-13 indicates minimal depression, 14-19 indicates mild depression, 20-28 indicates moderate depression and 29-63 indicates severe depression.

Depressive symptoms (Hamilton Depression Rating Scale)

The HDRS is a well-established and widely used rater-administered scale to measure the severity of depressive symptoms (Andrews & Williams, 2014; Hamilton, 1960; Williams, 1988). The scale consists of 17 items, which are grouped into different categories, such as depressed mood, suicide, insomnia, and somatic symptoms. Eight items are scored on a 5-point scale increasing in severity, where nine items are scored on a 3-point scale. The maximum score obtainable is 53. Scores of 0-17 indicates no depression, 8-13 indicates mild depression, 14-18 indicates moderate depression, 19-22 indicates severe depression, and above 23 indicates very severe depression.

6.2. Secondary outcome measures:

Beck Anxiety Inventory (BAI)

The BAI (A. T. Beck, Epstein, N., Brown, G., & Steer, R. A, 1988) is an inventory that measures the severity of anxiety, and was developed specifically to distinguish anxiety from depression. The inventory contains 21 items, which assess symptoms such as nervousness and fear of dying, where the items are rated on a 4-point Likert scale, which ranges from 0 (not at all) to 3 (severely). The maximum score is 63, where the cut-off point of clinical anxiety is 16 (Aaron T Beck & Steer, 1990). Higher scores on the BAI suggest a greater intensity of symptoms of anxiety. The BAI is viewed as an accurate measure of anxiety with high reliability and validity in non-clinical and clinical settings (Fydrich, Dowdall, & Chambless, 1992).

Meta-cognitive scales:

Major Depressive Disorder Scale (MDD-S)

MDD-S is a short self-report scale designed to measure depression levels, meta-cognitive beliefs and maladaptive coping-skills over the past week. The scale was developed as an assessment tool to evaluate baseline levels and progress in MCT treatment (Wells, 2009b).

Metacognitions Questionnaire (MCQ-30)

The MCQ 30 (Wells & Cartwright-Hatton, 2004) is a 30-item measure consisting of 5 subscales of beliefs which are: positive beliefs about worry, negative beliefs about thoughts concerning uncontrollability and danger, cognitive confidence, negative beliefs about the need to control thoughts, and cognitive self-consciousness (Wells & Cartwright-Hatton, 2004). Responses are rated on a 4-point Likert scale, which ranges from 1 (do not agree) to 4 (agree very much).. The scale has been found to have good construct and predictive validity and is responsive to treatment.

Negative and beliefs about rumination scales (NBRS)

The NBRS is a 13-item scale that measures negative beliefs about the uncontrollability and danger of ruminating (Papageorgiou & Wells, 2003). The scale consists of 13 statements, such as 'I will lose control if I ruminate too much' and 'ruminating makes me physically ill'. These statements are rated on a 4-point Likert scale ranging from 1 (do not agree) to 4 (agree very much). The NBRS show good internal consistency, validity and fit to the MCT model (Roelofs, Huibers, Peeters, Arntz, & van Os, 2010).

Positive beliefs about rumination scales (PBRS)

The PBRS was developed to measure positive beliefs about rumination (Papageorgiou & Wells, 2002). The self-report scale contains 9 items consisting of statements such as 'I need to ruminate about my problems to find answers to my depression'. Each stement is rated on a 4-point Likert scale ranging from 1 (do not agree) to 4 (agree very much). In studies examining the psychometric properties of the PBRS good internal consistency and validity have been demonstrated (Huibers et al., 2014; Roelofs et al., 2010)

Rumination Response Scale (RRS)

The RRS is a subscale of the Ruminative response questionnaire, RRQ (Nolen-Hoeksema & Morrow, 1991), and measures the response styles to depressed mood, which are focused on 'the self, on symptoms, and on possible causes and consequences of the mood. The scale consists of 22 items and contains statements such as 'think about how sad you feel' and is rated on a scale from 1 (almost never) to 4 (almost always). (Thanoi & Klainin-Yobas, 2015) tested the psychometric properties of the RRS on 747 undergraduates and found satisfactory reliability.

Cognitive scales:

Dysfunctional Attitude Scale (DAS)

The DAS is a scale that measures negative cognitions in depression, and examines the dysfunctional attitudes the individual has towards the self and the world (Weissman & Beck, 1978). The scale consists of 40 items, where each item is rated on a 7-point Likert scale ranging from 1 (fully disagree) to 7 (fully agree). The maximum score obtainable is 280, where higher scores indicate a higher intensity and presence of dysfunctional attitudes. Reliability and validity data for the DAS support its use as a measure of depressionogenic beliefs in an adult population (Oliver & Baumgart, 1985)

Young's Schema Questionnaire (YSQ-SF) - short version

The YSQ contains 16 subscales that measure early maladaptive schemas (Young & Brown, 1994), which contribute to perceptions of the self, the world and others (Rijkeboer, van den Bergh, & van den Bout, 2005). The short version consists of 75 items out of the 205 items from the long version, and measure schemas such as emotional deprivation, abandonment, and vulnerability to harm (Young & Brown, 1994). Each schema is rated on a 6-point scale ranging from 1 (completely untrue of me) to 6 (describes me perfectly).

Other measures:

Co-morbidity (SCID I)

The SCID-I (Spitzer et al., 1992) is a semi-structured interview to be administered by a clinician, and was used to determine presence of Axis I diagnoses. The semi-structured interview consists of an introduction and nine modules.

Patient-therapist alliance (Working Alliance Inventory, WAI)

The WAI is a self-report instrument used to assess the quality of alliance between the therapist and the patient and can be administered by the therapist and the patient (Adam O Horvath & Greenberg, 1989a). This 36-item inventory is based on Bordin's theory of therapeutic alliance (Bordin, 1979), which also constitutes the three subscales of the inventory, which are: goals; the agreement of goals between therapist and patient; tasks; the agreement of the tasks throughout therapy to reach these goals; and bond, the direct relationship between therapist and patient, which involve factors such as mutual trust (Tracey & Kokotovic, 1989). Each item is rated on a 7-point Likert scale, where the options range from 1 (never) to 7 (always). The inventory has also demonstrated sufficient validity (Adam O. Horvath & Greenberg, 1989b).

Patients' expectancies about treatment outcome

The Patient' expectancies about treatment outcome contains two questions, which ask about how logical the treatment seems to the patient during participation, and to what extent the patient feels the treatment will help them reduce their symptoms. Each question was rated on a 1-5 scale ranging from 'Not at all' to 'Very much'.

Objective measure (Concentration and attention subtests from the WAIS)

Wechslers Adult Intelligence Scale (WAIS) is one of the most widely used instruments to measure intelligence and cognitive functioning (Kaufman & Lichtenberger, 2005). The instrument contains a range of subtests that measure dimensions such as verbal comprehension, perpetual reasoning, working memory and processing speed (Wechsler, 2014)). This measure is included for analyses in a separate study and not for the main trial.

7. Ethical Considerations

The present study uses the best-proven standards for CBT and MCT. All therapists are experienced clinical psychologists working under the Nordic Ethical Guidelines and according to good clinical

practice standards described at Dansk Psykolog Forening (www.dp.dk). The trial design and consent form was formally approved by the Danish ethics committee on the 13th of December 2010. All participants considered for the trial will be provided with written and oral information so that they can make an informed decision about participation.
This trial will be conducted in compliance with the protocol approved by Manchester University and the Danish Ethics Committee (www.cvk.im.dk/cvk/).

8. Safety Reporting and Adverse events

An adverse event can be defined as:

Any unfavourable, unintended effect that occurs during a study (e.g. symptom, diagnosis, test finding), having been absent at baseline, or if present at baseline appears to worsen.

Information related to an adverse event will be captured in the case file and will be regularly monitored by the research management group in line with Good Clinical Practice Guidelines. In the case of adverse events like suicide attempts or sudden accidents it was decided to take action and report and exclude patients from the trial in order to get them hospitalised. However, no adverse events were reported in this trial.

A serious adverse event (SAE), is any untoward medical occurrence that

- results in death
- is life threatening (including suicide attempts)
- requires hospitalisation
- results in persistent or significant disability/incapacity

SAE's will be reported to the ethical committee that gave a favourable opinion to the study where in the opinion of the chief investigators the event was related to the research procedures. The response to SAE's will be determined on a case by case basis and will be in line with local policies and procedures.

9. Trial design and analysis

The trial is a single blind randomised, parallel between-groups design with 6-months follow-up (See trial flow diagram and timeline [Trial Registration Number: ISRCTN82799488 - Meta-cognitive Therapy (MCT) versus Cognitive Behaviour Therapy (CBT) for Depression. An assessor will undertake HRSD ratings and will remain blind to treatment allocation. Randomisation lists will be drawn up independently and held outside of the study team.

Severity of depression and gender of participants will be used as stratification variables during randomization. Participants will be assigned to therapists within treatment modality based on therapist availability.

Patients will be allocated up to a maximum of 24 sessions of MCT or CBT and assessed at pre-treatment, mid-treatment, post-treatment and at 6-months follow-up.

9.1. Sample size

There are currently no direct comparisons of MCT against CBT for depression to inform sample size estimates. Therefore we plan the current study to detect a post-treatment difference between groups based on mean BDI-II scores observed in previous studies that tested MCT or CBT separately (Dimidjian, Hollon, Dobson et al, 2006; Papageorgiou & Wells, 2015). The mean following MCT was 10 and the mean for CBT 14, suggesting a group difference of 4 points (a standardized effect size of 0.57), we used 7 as the standard deviation (Papageorgiou & Wells, 2015). Using an alpha of 5% and 90% power, this indicated that 64 participants per group were required (total n=128). We plan to increase the target sample by a further 20% to allow for pre-to-post treatment attrition.

9.2. Randomisation and Data Analysis

Randomisation will occur after initial pre-treatment evaluation by an external and independent person who holds a secret randomisation list produced by Manchester University. This list will be stratified for severity of depression (high/low) and gender (males/females). Scores on pre-treatment HDRS will be used to form four groups: 1) high severity (HDRS $\geq$ 20), 2) low severity (HDRS $\leq$ 19), 3) males and 4) females. Participants will be assigned to the two therapists (PC and SF) based on therapist availability.

A statistical Analysis Plan will be drawn up before study completion and the final data will be analysed by independent trial statisticians at The University of Manchester department of bio-statistics.

10. Blinding

All assessments will be administered by an independent assessor: Charlotte Koch Andersen (CKA) CKA is a clinical psychologist with over 10 years of experience in therapy and assessment. This is a blinded study and all patients will be instructed not to reveal which treatment they have received at post-assessment and follow-up. In order to control for evaluation biases the independent assessor CKA will be asked to guess which treatment the patient has received at post-treatment assessment.

11. Interventions

In this study, protocols for CBT and MCT for MDD will be compared. These treatment manuals include the following elements:

11.1. Cognitive behaviour Therapy (CBT)

The CBT group receive treatment using Beck’s traditional cognitive therapy (A. T. Beck, Rush, A.J., Shaw, B. F., & Emery, G. , 1979) facilitated by Melanie Fennel’s depression protocol from (*Cognitive behaviour therapy for psychiatric problems : a practical guide / Keith Hawton ... [et al.]*, 1989).

- an agreed ‘problem-goal’-list
- 4-page booklet called "Coping with depression"
- negative automatic thought restructuring
- schema-related restructuring
- activity scheduling planned throughout treatment and level of mastery and pleasure increased
- relapse prevention

11.2. Meta-cognitive therapy for depression (MCT)

Meta-cognitive Therapy will follow the Wells, (2009b) manual and consists of:

- practise of Attention Training
- detached mindfulness practise
- challenging negative metacognitive beliefs
- challenging positive metacognitive beliefs
- relapse prevention

12. Competency and treatment adherence

In order to establish a fair and even level of competency and experience in CBT and MCT 24 patients were initially recruited to a pilot study (n=12 CBT, n=12 MCT) as training cases and not entered into the trial.

Treatment adherence and competency in both treatments is of vital importance. Highly experienced clinical psychologists PC and SF will deliver therapy. PC and SF have undergone specialist training and supervision in CBT with a minimum of ten years of clinical experience in the field. Leading senior psychologist and CBT therapist Lennart Holm, with 35 years of CBT experience, will supervise the CBT condition and Professor Adrian Wells, the originator of MCT, will supervise the MCT treatment to ensure both treatments are delivered optimally and according to protocol.

All sessions will be recorded on videotape and screened by external psychologists Sisse Find Nielsen (SFN) and Anny Lee (AL) for potential contamination of methods. Contamination is defined by the extent of 'object-level' work done in the MCT condition or 'meta-level' work done in the CBT condition..

20 random videotaped sessions (10 MCT and 10 CBT) will be selected and rated by final-year postgraduate psychology students for treatment adherence using the treatment fidelity checklists for CBT and MCT. To add an extra layer of quality checking to the CBT arm 10% of CBT tapes will be transcribed, translated and sent to external experts for evaluation of competency.

13. References

Andrews, G., & Williams, A. D. (2014). Internet psychotherapy and the future of personalized treatment. *Depression and Anxiety, 31*(11), 912-915.

Beck, A. T., Epstein, N., Brown, G., & Steer, R. A. (1988). An inventory for measuring clinical anxiety: Psychometric properties. . *Journal of Consulting and Clinical Psychology, 56*, 893–889.

Beck, A. T., Rush, A. J., Shaw, B. F., & Emery, G. (1979). *Cognitive therapy of depression*. New York: Guilford.

Beck, A. T., Rush, A.J., Shaw, B. F., & Emery, G. . (1979). *Cognitive therapy of depression*. New York: Guilford.

Beck, A. T., & Steer, R. A. (1990). Manual for the Beck anxiety inventory.

Beck, A. T., Steer, R.A., Ball, R., & Ranieri, W. (1996). Comparison of Beck Depression Inventory -IA and -II in psychiatric outpatients *Journal of Personality Assessment, 57*, 588–597.

Beck, A. T., Ward, C., & Mendelson, M. (1961). Beck depression inventory (BDI). *Arch Gen Psychiatry, 4*(6), 561-571.

Callesen, P., Jensen, A. B., & Wells, A. (2014). Metacognitive therapy in recurrent depression: A case replication series in Denmark. *Scandinavian Journal of Psychology, 55*(1), 60-64. doi:10.1111/sjop.12089

Cristea, I. A., Huibers, M. J. H., David, D., Hollon, S. D., Andersson, G., & Cuijpers, P. (2015). The effects of cognitive behavior therapy for adult depression on dysfunctional thinking: A meta-analysis. *Clinical Psychology Review, 42*, 62-71. doi:10.1016/j.cpr.2015.08.003

Cuijpers, P., Andersson, G., Donker, T., & van Straten, A. (2011). Psychological treatment of depression: Results of a series of meta-analyses. *Nordic Journal of Psychiatry, 65*(6), 354-364.

Cuijpers, P., Geraedts, A. S., van Oppen, P., Andersson, G., Markowitz, J. C., & van Straten, A. (2011). "Interpersonal psychotherapy for depression: A meta-analysis": Correction. *The American Journal of Psychiatry, 168*(6), 652.

Dammen, T., Papageorgiou, C., & Wells, A. (2015). An open trial of group metacognitive therapy for depression in Norway. *Nordic Journal of Psychiatry, 69*(2), 126-131. doi:10.3109/08039488.2014.936502

Dimidjian, S., Hollon, S. D., & Dobson, K. S. (2006). Randomized trial of behavioural activation, cognitive therapy, and anti-depressant medication in the acute treatment of adults with major depression. . *Journal Of Consulting And Clinical Psychology, 74*, 658-670.

Driessen, E., Hegelmaier, L. M., Abbass, A. A., Barber, J. P., Dekker, J. J. M., Van, H. L., . . . Cuijpers, P. (2015). The efficacy of short-term psychodynamic psychotherapy for depression: A meta-analysis update. *Clinical Psychology Review, 42*, 1-15. doi:10.1016/j.cpr.2015.07.004

Fydrich, T., Dowdall, D., & Chambless, D. L. (1992). Reliability and validity of the Beck Anxiety Inventory. *Journal of Anxiety Disorders, 6*(1), 55-61.

Gotink, R. A., Chu, P., Busschbach, J. J., Benson, H., Fricchione, G. L., & Hunink, M. (2015). Standardised mindfulness-based interventions in healthcare: An overview of systematic reviews and meta-analyses of RCTs. *PLoS ONE Vol 10(4), Apr 2015, ArtID e0124344, 10*(4).

Groves, S. J., Porter, R. J., Jordan, J., Knight, R., Carter, J. D., McIntosh, V. V. W., . . . Joyce, P. R. (2015). Changes in neuropsychological function after treatment with metacognitive therapy or cognitive therapy for depression. *Depression And Anxiety, 32*(6), 437-444. doi:10.1002/da.22341

Hagen R, Hjemdal O, Solem S, Kennair LEO, Nordahl HM, Fisher P and Wells A (submitted) Metacognitive Therapy for Depression in Adults: A Waiting List Randomized Controlled Trial with Six Months Follow-Up. Front. Psychol. 8:31.

Hamilton, M. (1960). A rating scale for depression. *Journal of Neurology, Neurosurgery, and Psychiatry, 23,* 56–62.

Horvath, A. O., & Greenberg, L. S. (1989a). Development and validation of the Working Alliance Inventory. *Journal of Counseling Psychology, 36*(2), 223.

Horvath, A. O., & Greenberg, L. S. (1989b). Development and Validation of the Working Alliance Inventory. *Journal of Counseling Psychology, 36*(2), 223-233.

Huibers, M. J. H., van Breukelen, G., Roelofs, J., Hollon, S. D., Markowitz, J. C., van Os, J., . . . Peeters, F. (2014). Predicting response to cognitive therapy and interpersonal therapy, with or without antidepressant medication, for major depression: A pragmatic trial in routine practice. *Journal Of Affective Disorders, 152*, 146-154. doi:10.1016/j.jad.2013.08.027

Jacobson, N. S., & Truax, P. (1991b). Clinical significance: A statistical approach to defining meaningful change in psychotherapy research. *Journal of Consulting and Clinical Psychology, 59*(1), 12-19. doi:10.1037/0022-006X.59.1.12

Johnsen, T. J., & Friborg, O. (2015). The effects of cognitive behavioral therapy as an anti-depressive treatment is falling: A meta-analysis. *Psychological Bulletin, 141*(4), 747. doi:10.1037/bul0000015

Jordan, J., Carter, J. D., McIntosh, V. V., Fernando, K., Frampton, C. M., Porter, R. J., . . . Joyce, P. R. (2014). Metacognitive therapy versus cognitive behavioural therapy for depression: a randomized pilot study. *Australian and New Zealand Journal of Psychiatry,* 0004867414533015.

Nolen-Hoeksema, S., & Morrow, J. (1991). A prospective study of depression and posttraumatic stress symptoms after a natural disaster: the 1989 Loma Prieta Earthquake. *Journal of personality and social psychology, 61*(1), 115.

Oliver, J., & Baumgart, E. (1985). The Dysfunctional Attitude Scale: Psychometric properties and relation to depression in an unselected adult population. *Cognitive Therapy And Research, 9*(2), 161-167. doi:10.1007/BF01204847

Papageorgiou, C., & Wells, A. (2002). Positive beliefs about depressive rumination: Development and preliminary validation of a self-report scale. *Behavior Therapy, 32*(1), 13-26.

Papageorgiou, C., & Wells, A. (2003). An empirical test of a clinical metacognitive model of rumination and depression. *Cognitive Therapy and Research, 27*(3), 261-273.

Papageorgiou, C., & Wells, A. (2004). *Depressive rumination: Nature, theory and treatment*: John Wiley & Sons.

Papageorgiou, C., & Wells, A. (2015). Group Metacognitive Therapy for Severe Antidepressant and CBT Resistant Depression: A Baseline-Controlled Trial. *Cognitive Therapy and Research, 39*(1), 14-22. doi:10.1007/s10608-014-9632-x

Rijkeboer, M. M., van den Bergh, H., & van den Bout, J. (2005). Stability and discriminative power of the Young Schema-Questionnaire in a Dutch clinical versus non-clinical population. *Journal of Behavior Therapy and Experimental Psychiatry, 36*(2), 129-144.

Roelofs, J., Huibers, M., Peeters, F., Arntz, A., & van Os, J. (2010). Positive and Negative Beliefs About Depressive Rumination: A Psychometric Evaluation of Two Self-Report Scales and a Test of a Clinical Metacognitive Model of Rumination and Depression. *Cognitive Therapy And Research, 34*(2), 196-205. doi:10.1007/s10608-009-9244-z

Spitzer, R. L., Williams, J. B., Gibbon, M., & First, M. B. (1992). The structured clinical interview for DSM-III-R (SCID): I: history, rationale, and description. *Archives of General Psychiatry, 49*(8), 624-629.

Thanoi, W., & Klainin-Yobas, P. (2015) Assessing rumination response style among undergraduate nursing students: A construct validation study. *Nurse Education Today, 35*(5), 641-646. doi:10.1016/j.nedt.2015.01.001

Wechsler, D. (2014). Wechsler Adult Intelligence Scale–Fourth Edition (WAIS–IV).

Weissman, A. N., & Beck, A. T. (1978). Development and validation of the Dysfunctional Attitude Scale: A preliminary investigation.

Wells, A. (1990). Panic disorder in association with relaxation induced anxiety: An attentional training approach to treatment. *Behavior Therapy, 21*(3), 273-280.

Wells, A. (1995). Metacognition and worry - A cognitive model of GAD. *Behavioural and Cognitive Psychotherapy, 23*, 20.

Wells, A. (2002). Worry, Metacognition and GAD. *Journal of Cognitive Psychotherapy, 16*(2), 179-192.

Wells, A. (2005a). Detached Mindfulness In Cognitive Therapy: A Metacognitive Analysis And Ten Techniques. *Journal of Rational-Emotive and Cognitive-Behavior Therapy, 23*(4), 337-355. doi:10.1007/s10942-005-0018-6

Wells, A. (2005b). Worry, Intrusive Thoughts, and Generalized Anxiety Disorder: The Metacognitive Theory and Treatment. In D. A. Clark (Ed.), *Intrusive Thoughts in Clinical Disorders - Theory, Research and Treatment* (pp. 119-144). (25 NP) New York: The Guilford Press.

Wells, A. (2009a). *Meta-cognitive Therapy for depression and anxiety*. UK, The Guilford Press, London: (127 NP) New York: Guilford Press.

Wells, A. (2009b). *Metacognitive therapy for anxiety and depression / Adrian Wells*. New York: New York : Guilford.

Wells, A., & Matthews, G. (1996a). Modelling Cognition in Emotional Disorder - The S-REF Model *Behavior Research and Therapy, 34*(11/12), 881-888.

Wells, A., & Matthews, G. (1996b). Modelling cognition in emotional disorder: The S-REF model. *Behaviour Research and Therapy, 34*(11), 881-888.

Wells, A., & Matthews, G. (2014). *Attention and Emotion (Classic Edition): A Clinical Perspective*: Psychology Press.

Wells, A., & Cartwright-Hatton, S. (2004). A short form of the metacognitions questionnaire: properties of the MCQ-30. *Behaviour Research and Therapy, 42*(4), 385-396.

Wells, A., Fisher, P., Myers, S., Wheatley, J., Patel, T., & Brewin, C. R. (2012). Metacognitive therapy in treatment-resistant depression: A platform trial. *Behaviour research and therapy, 50*(6), 367-373. doi:10.1016/j.brat.2012.02.004

Young, J. E., & Brown, G. (1994). Young schema questionnaire. *Cognitive therapy for personality disorders: A schema-focused approach*, 63-76.

Appendix 1: Study Flow Chart

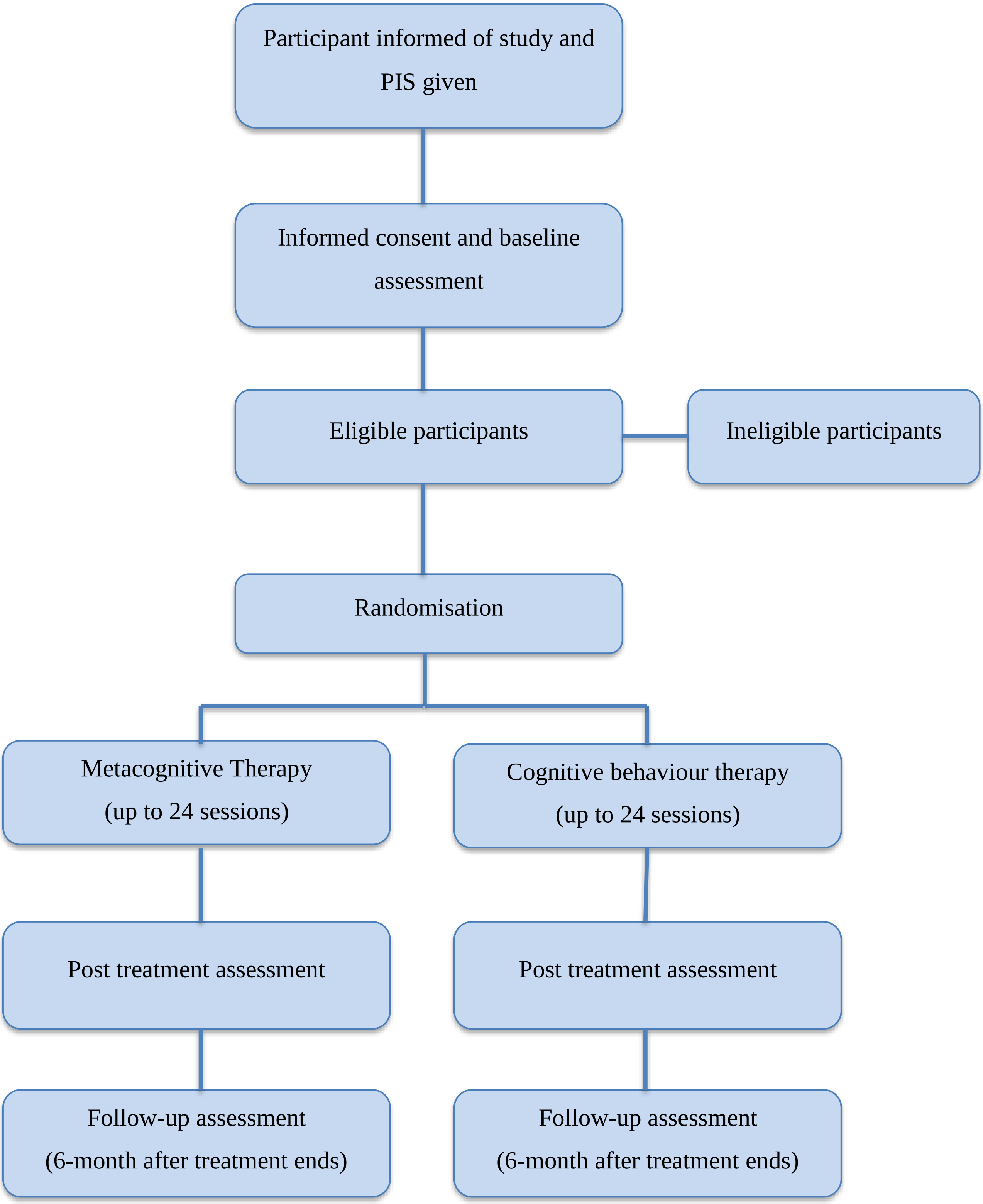

Appendix 2: Schedule of Measurement

Measure	T1 Pre	Sessional	T2 Post	T3 FU
Primary:				
BDI-II	X	X	X	X
HDRS	X		X	X
Secondary:				
BAI	X		X	X
MCQ-30	X		X	X
PBRS	X		X	X
NBRS	X		X	X
RRS*	X		X	X
DAS	X		X	X
YSQ	X		X	X

BDI-II: Beck Depression Inventory

HDRS: Hamilton Rating Scale for Depression

BAI: Beck Anxiety Inventory

MCQ-30: Metacognitions Questionnaire 30 Item

PBRS: Positive Beliefs about Rumination Scale

NBRS: Negative Beliefs about Rumination Scale

RRS*: Ruminative Response Styles Questionnaire

DAS: Dysfunctional Attitudes Scale (A)

YSQ: Young's Schema Questionnaire

**Note: Due to a printing error the RRS measure was corrupted and therefore dropped from the study analysis.*
